# Physical and physiological demands in elite female U-19 basketball: position-specific and competitive level differences in North African players

**DOI:** 10.7717/peerj.21218

**Published:** 2026-06-10

**Authors:** Nidhal Ben Abdlekrim, Mohamed Ali Nabli, Wissem Dhahbi, Halil İbrahim Ceylan, Valentina Stefanica, Mohamed Romdhani, İsmail Dergaa, Karim Chamari

**Affiliations:** 1 Higher Institute of Sport and Physical Education, ISSEP Ksar Saïd, Manouba University, Tunis, Tunisia; 2 Tunisian Research Laboratory “Sports Performance Optimization”, National Center of Medicine and Science in Sports (CNMSS), Tunis, Tunisia; 3 Research Unit “Sport Sciences, Health and Movement”, High Institute of Sports and Physical Education of Kef, University of Jendouba, Kef, Tunisia; 4 Qatar Police Academy, Police College, Training Department, Doha, Qatar; 5 Physical Education and Sports Teaching Department, Faculty of Sports Sciences, Ataturk University, Erzurum, Turkey; 6 Department of Physical Education and Sport, Faculty of Sciences, Physical Education and Informatics, National University of Science and Technology Politehnica Bucharest, Pitesti University Center, Pitesti, Romania; 7 Interdisciplinary Laboratory in Neurosciences, Physiology and Psychology: Physical Activity, Health and Learning (LINP2), UFR STAPS (Faculty of Sport Sciences), Paris Nanterre University, Nanterre, France; 8 Physical Activity, Sport and Health, National Observatory of Sports, Tunis, Tunisia; 9 High Institute of Sport and Physical Education of Ksar Said, University of Manouba, Manouba, Tunisia; 10 Naufar, Wellness and Recovery Center, Doha, Qatar

**Keywords:** Anaerobic threshold, Physiological response, Basketball, Time-motion analysis, Youth athletes, Sport-specific training

## Abstract

**Aim:**

Understanding the physical and physiological demands of female youth basketball is essential for optimizing training and performance monitoring. However, evidence describing match demands in elite U-19 female players, particularly in African contexts, remains limited. Existing profiles are largely derived from male or adult cohorts and may not accurately reflect youth competition. This study aimed to examine the physical and physiological demands of elite North African U-19 female basketball players, considering differences by playing position (guards, forwards, centers) and competitive level (national *vs.* international).

**Methods:**

Thirty elite Tunisian U-19 female players (age 18.3 ± 0.2 years, height 1.78 ± 0.05 m, mass 82.9 ± 4.8 kg; 15 national-level, 15 international-level) were monitored during eight playoff games. Video-based time-motion analysis quantified activity frequency and duration across nine movement categories (standing, walking, jogging, running, sprinting, jumping, low/moderate/high-intensity shuffling). Physiological responses included heart rate (HR) monitoring (four intensity zones: <75%, 75–85%, 85–95%, >95% HRmax) and capillary [La] sampling. Two-way ANOVA (position × level) examined main and interaction effects.

**Results:**

International-level players performed significantly more high-intensity activities than national-level players (224.0 ± 5.1 *vs.* 214.1 ± 5.4; *p* < 0.001, *d* = 1.95) and spent more time in maximal HR zones (16.1 ± 0.3% *vs.* 12.1 ± 0.3%; *p* < 0.001, *d* = 13.33), indicating greater fatigue resistance. Guards executed more high-intensity shuffling actions than forwards and centers (*p* < 0.001, *η*^2^ = 0.92), whereas centers performed more static high-intensity actions and exhibited higher [La] concentrations (5.22 ± 0.15 *vs.* 4.93 ± 0.13 and 4.64 ± 0.12 mmol L^−1^; *p* < 0.001, *η*^2^ = 0.76). High-intensity activity declined from the first to the fourth quarter in both groups (*p* < 0.001, *η*^2^ = 0.94), with a greater reduction in national-level players (37.0% decline) than international players (31.9% decline). Intra-observer reliability was excellent across all movement categories (ICC ≥ 0.91; CV ≤ 4.6%).

**Conclusion:**

U-19 female basketball imposes distinct position-specific demands (guards: high-intensity lateral movements; centers: static exertions and elevated metabolic load) and competitive-level differences (international players: superior fatigue resistance). These findings provide the first quantitative profile of elite African female youth basketball, establishing reference benchmarks for position-specific conditioning and competitive-level progression assessment.

## Introduction

Developing a sport-specific conditioning program requires an understanding of the demands placed on players during matches (Scanlan et al., 2012). The most significant benefits are achieved when the training stimulus mimics or exceeds the intensity of the competition. This paradigm is thought to provide physiological adaptations that enhance maximal performance.

However, while the activity profile of U-19 men’s basketball is well characterized (Scanlan et al., 2012; Willberg, Kohler & Zentgraf, 2022), information on game intensity during current U-19 women’s basketball competitions is relatively scarce (*i.e.,* very limited research has been conducted). Physical training for female players is often derived from men’s basketball, although significant gender differences in game activities have been previously noted (Scanlan et al., 2012). Furthermore, the physical and physiological demands of female basketball (*i.e.,* Australian and Spanish) have primarily focused on adult players (Willberg, Kohler & Zentgraf, 2022; Huynh et al., 2024). Recent methodological advances in basketball demand profiling have emphasized contextual variability in physical performance metrics, including peak demands across different time epochs and the influence of playing position, competitive level, and match phase on external load variables (Sosa et al., 2021; Fox, Stanton & Scanlan, 2018). Studies using local positioning systems have established age-specific absolute velocity thresholds that differ across competitive categories (Sosa et al., 2021), demonstrating that physical demand profiles are not uniform but vary systematically with player maturation and competitive context. However, no published research has examined elite U-19 female basketball in African populations, where physical characteristics, tactical approaches, and competitive structures may differ from European and Australian contexts (Lockie et al., 2020). Furthermore, existing female basketball research has examined either physical demands or physiological responses in isolation, without concurrent assessment across playing positions, competitive levels, and game phases. These findings underscore the need to establish population-specific reference values rather than extrapolating from adult or male cohorts when characterizing the demands of female youth basketball. A previous study (Power et al., 2022) examined the effect of positional role and competition level (*i.e.,* national *vs.* international) on game intensity in European basketball. They reported higher blood lactate concentrations ([La]) in guards than in forwards and centres within the international group; however, no differences were observed between the national players’ levels (NPL). Heart-rate (HR) responses showed a similar positional difference, with higher HRs in guards than in forwards and centres. On the other hand, HR among international-level players (ILP) was higher than that of non-professional-level players (NLP). Indeed, a detailed description of players’ movement patterns throughout the game seems crucial in developing specific fitness programs and tests for female U-19 basketball players.

Female basketball comprises short explosive actions (∼4.2 s) interspersed with active recovery periods (jogging: ∼35.6 s; running: ∼16.7 s) (Scanlan, Dascombe & Reaburn, 2011), with activity profiles differing substantially between elite and sub-elite players (Willberg, Kohler & Zentgraf, 2022). However, existing energy system and activity pattern data derive from adult or male populations, limiting applicability to female youth players where physiological maturation, tactical sophistication, and competitive structures differ (Arede et al., 2019).

Time-motion analysis results for European female players (Lockie et al., 2020) revealed that guards made more movements and sprints than players in other positions. Furthermore, forwards and centres performed significantly more jumps and static exertion (*i.e.,* picking) than guards. But these details are indicative of female European games. They may not be pertinent to female basketball games in African contexts, given that players’ movement-specificities and physical characteristics vary systematically across geographic regions (Lockie et al., 2020). However, despite basketball’s popularity among teenagers, very limited research has examined the physical loads imposed on U-19 female players during official games.

Despite the global growth of female basketball, concurrent assessment of physical activity patterns (movement frequency, intensity distribution) and physiological responses (HR, [La]) across playing positions, competitive levels, and game quarters remains absent in U-19 female populations, particularly in African contexts. This study quantified position-specific (guards, forwards, centers) and level-specific (national *vs.* international) differences in activity demands and physiological responses during elite North African U-19 female basketball competition to establish reference profiles for this underexamined population.

## Materials & Methods

### Design

The experiment was performed during the play-off stage of the U-19 Tunisian female basketball championship. Eight games played over one week involving four different teams were analyzed. All games were played on Sunday at 11:00 under similar environmental conditions (25−28 °C, 55% humidity). Each game consisted of four 10-minute quarters, with a 10-minute halftime interval and a 2-minute break between quarters of each half.

Players’ physical demands were assessed using video time-motion analysis (Ben Abdelkrim et al., 2010b; Ben Abdelkrim, El Fazaa & El Ati, 2007). Physiological game requirements were assessed by monitoring HR, and [La] (Ben Abdelkrim et al., 2010a; Ben Abdelkrim et al., 2009). Each player was assessed during a single complete game, with no repeated measurements across multiple games, ensuring independent observations for statistical analysis. The live-time and total-time for each player were then calculated (Ben Abdelkrim, El Fazaa & El Ati, 2007). Twenty minutes before the game started, players completed a standardized warm-up on the court. The study followed a cross-sectional design with two categorical independent variables: playing position (three levels: guards, forwards, centers) and competitive level (two levels: national and international). Four blood samples were collected from each participant throughout a game, immediately upon completion of the quarter.

### Participants

The study protocol received approval from the Ethics Committee of the High Institute of Sports and Physical Education of Kef, University of Jendouba, Tunisia (approval number: C-0008/2024) and adhered to the Declaration of Helsinki principles. All participants and their legal guardians provided written informed consent after receiving detailed verbal and written explanations of study procedures and potential risks.

Thirty Tunisian U-19 female basketball players, representing four teams from the 1st national division, were studied during eight separate games. They were starters (*i.e.,* major players of their teams) performing in the same national U-19 basketball championship. The sample includes 15 NLP (age 18.31 ± 0.23 years, height 1.78 ± 0.06 m, weight 82.70 ± 4.30 kg, body fat 16.45 ± 4.90% and estimated V̇O_2_max 50.59 ± 1.26 ml  kg^−1^ min^−1^) and 15 ILP (age 18.23 ± 0.22 years, height 1.79 ± 0.04 m, weight 83.10 ± 5.40 kg, body fat 15.4 ± 3.11% and estimated V̇O_2_max 53.73 ± 1.36 ml  kg^−1^.min^−1^). ILP were defined as players selected for national team representation in FIBA-sanctioned U-19 international competitions (AfroBasket U-18 Women, FIBA U-19 Women’s Basketball World Cup qualifying tournaments). In contrast, NLP competed exclusively at the national championship level without international selection (Arede et al., 2019).

One week before the games, participants’ heights were measured to the nearest 0.1 cm using a portable stadiometer (Holtain Ltd, Crymych, United Kingdom). Body mass was obtained to the nearest 0.1 kg using an electronic scale (Seca Instruments Ltd, Hamburg, Germany). Skinfold thickness at four sites (biceps, triceps, sub-scapular, and supra-iliac) was measured using a Harpenden caliper (Lange, Cambridge, MA, USA), and from these measurements, %body-fat was estimated (Silveira et al., 2020). Maximal aerobic power (*i.e.,* power relative to V̇O_2_max) was calculated using the Yo-Yo intermittent recovery test level 1, with distance covered as described by Castagna et al. (2008).

Video recordings were obtained using a fixed camcorder (Sony, model HVR-HD 1000E, Japan) mounted on a tripod, positioned ∼12 m from the sideline at half-court, at a height of ∼20 m to allow full-court coverage. The software adopted (PC-foot 4) enabled the detection of players’ positions in each frame (Zhang et al., 2025). The video footage was analysed frame by frame with an accuracy of 0.04 s. Nine movement groups were used to categorize the form and intensity of game activity, including standing, walking, jogging, running, sprinting, jumping, and low-, moderate-, and high-intensity shuffling movements (Zhang et al., 2025). Additionally, two states of static-exertion (picks and positioning) were included (Ben Abdelkrim et al., 2010b). Shuffling movements account for 18–42% of game time in female basketball (Taylor et al., 2017) and require frontal-plane stability and eccentric control, with sex-specific neuromuscular control patterns documented during multidirectional movements (Leidersdorf et al., 2022; Chaudhari et al., 2007). Movement categories were grouped by intensity: high-intensity (sprinting > 7 m s^−1^, jumping, high-intensity shuffling > 2 m s^−1^ in defensive stance, static exertions (picks/positioning)); moderate-intensity (running 3.1−7.0 m s^−1^, moderate shuffling 1.67−2.5 m s^−1^); low-intensity (jogging 1.1−3.0 m s^−1^, low shuffling ≤1.67 m s^−1^); passive recovery (standing <1 m s^−1^); active recovery (walking 1.0−1.8 m s^−1^). These thresholds align with established female basketball movement classifications (Sosa et al., 2021; Reina, García-Rubio & Ibáñez, 2020). The frequency of bouts and the lifetime spent in each movement category were calculated. Live time included the game time during which the game clock was running and the player was on the court, as well as those short durations when the player was active during out-of-bounds situations. Total time is considered to be the duration that a player was on the court, including all stoppages in play, excluding timeouts and stoppages unrelated to active play (Ben Abdelkrim, El Fazaa & El Ati, 2007). The reliability of the time-motion analysis procedures used in this study was assessed according to Ben Abdelkrim et al. (2010a) (Table 1) and conducted by a single person. The approach to establish reliability followed similar methodological principles to those in other sports movement assessment protocols (Padulo et al., 2019).

**Table 1:** Baseline characteristics and inter-phase variability metrics by latent class. Intra-observer reliability results for the time-motion analysis of basketball-specific movement categories. Reliability is reported for both movement frequency and time spent in each category using intraclass correlation coefficients (ICC) and coefficients of variation (CV%). Higher ICC values and lower CV values indicate excellent relative and absolute reliability of the measurement procedures.

**Movement category**	**Frequency**		**Time**	
	ICC	CV (%)	ICC	CV (%)
Standing	0.97	1.2	0.92	2.1
Walking	0.96	1.3	0.93	1.9
Jogging	0.95	1.5	0.95	1.6
Running	0.93	1.8	0.94	1.7
Sprinting	0.98	1.2	0.96	1.5
Low-intensity shuffling	0.92	2.3	0.93	2
Moderate-intensity shuffling	0.91	2.4	0.94	1.8
High-intensity shuffling	0.97	1.1	0.97	1.2
Jumping	0.93	4.6	0.95	3.2
Static exertion	0.96	2.5	0.96	2.1

**Notes.**

ICC intraclass correlation coefficient CV coefficient of variation

Movement categories were defined as: Standing <1 m s^−1^, Walking 1.0−1.8 m s^−1^, Jogging 1.1−3.0 m s^−1^, Running 3.1−7.0 m s^−1^, Sprinting >7.0 m s^−1^, Low-intensity shuffling ≤1.67 m s^−1^ (defensive stance), Moderate-intensity shuffling 1.67−2.5 m s^−1^ (defensive stance), High-intensity shuffling > 2.0 m s^−1^ (defensive stance), Jumping (take-off to landing), Static exertions (picks/positioning).

Game-HR was monitored at 5-second intervals using short-range telemetry (Polar S810 NV, Kempele, Finland). The HR monitor stopwatch was synchronized with the starting time before the game (Ben Abdelkrim, El Fazaa & El Ati, 2007; Ferioli et al., 2020). To determine the mean HR during live time, data for time stoppages (time-outs, free throws, and substitutions) were excluded. Relative-time was thus divided into four intensity zones: maximal (>95%HRmax), high (85–95%HRmax), moderate (75-<85%HRmax), and low (<75%HR_max_) (Dhahbi et al., 2024). HR_max_ was the highest reached heart rate during gameplay or the Yo-Yo IR1 test (Ben Abdelkrim, El Fazaa & El Ati, 2007; Ferioli et al., 2020).

To determine [La]s during the games, a blood sample was collected from the player’s fingertip within 1 min of the player’s rest period.

### Statistical analyses

Sample size was determined *a priori* using G*Power 3.1.9.7 for a two-way ANOVA (3 ×2 factorial design). Assuming large main effects (*f* = 0.40) based on previous research (Scanlan, Dascombe & Reaburn, 2011), *α* = 0.05, and power=0.85, a minimum *n* = 30 (*n* = 5 per subgroup) was required, consistent with basketball time-motion studies (*n* = 8–20) (Scanlan et al., 2012).

Data are expressed as mean±standard-deviation. Before performing ANOVAs, all variables were tested for normality using the Shapiro–Wilk test and homogeneity of variance using Levene’s test. A two-way analysis of variance (ANOVA) was employed, with playing position (guards, forwards, centers) and competitive level (NLP, ILP) as fixed factors, to examine main effects and potential interactions on physical and physiological metrics. Quarterly comparisons employed a mixed-design ANOVA with quarter (Q1–Q4) as the within-subjects factor and playing position and competitive level as between-subjects factors. Mauchly’s test of sphericity was applied to all within-subjects effects; where violated, degrees of freedom were corrected using the Greenhouse-Geisser epsilon. The F-statistics and degrees of freedom reported for quarterly effects (*e.g.*, F(3,84)) reflect this mixed-model structure. This approach was selected to control for Type I error inflation that could occur with multiple separate analyses (Batterham & Hopkins, 2006). The *η*^2^ (Eta Squared) was calculated to determine the magnitude of differences, interpreted as: small 0.01∼0.06; 0.06∼0.14 medium; large >0.14 (Batterham & Hopkins, 2006). Tukey’s *post-hoc* tests were conducted to determine specific between-group differences when significant main effects were identified. For significant interaction effects, simple main effects analyses were performed. Effect sizes for pairwise comparisons were calculated using Cohen’s d, interpreted as: trivial: <0.2; small: 0.2∼< 0.5; moderate: 0.5∼≤0.8; large: >0.8 (Sullivan & Feinn, 2012). The reliability of the time spent in each movement category and its frequency was determined. The Intra-class-coefficient (ICC) was calculated for the relative reliability (*i.e.,* poor for ICC < 0.5; moderate for 0.5<ICC < 0.75; good for 0.75 <  ICC < 0.9, and excellent for ICC≥0.90) (Koo & Li, 2016). The error technique was applied to achieve absolute reliability. This method calculates the coefficient of variation (CV%) for the differences between repeated measurements. Values below 10% indicate good absolute reliability, consistent with established thresholds for sports performance testing (Hopkins, Schabort & Hawley, 2001). This approach to reliability assessment follows established protocols for determining absolute and relative reliability in sports performance testing (Čular et al., 2021). The statistical package SPSS 19 (SPSS, Inc., Chicago, IL, USA) was used for statistical analyses. The significance level was set at *p* < 0.05. Given the small subgroup size (*n* = 5) and single-game observation design, the magnitude of reported effect sizes may be influenced by regression-to-the-mean effects and limited sampling variability (Barnett, vander Pols & Dobson, 2005). Large *η*^2^ values reflect the low within-group variance characteristic of categorical video-coded data with high observer reliability (ICC >  0.91), while extreme Cohen’s d values in HR zone comparisons arise from near-zero within-group variance in telemetry-derived aggregates rather than model misspecification. Consequently, effect size interpretation prioritizes descriptive characterization of observed differences rather than predictive generalization to broader populations. Confidence intervals for pairwise d values are reported alongside all primary comparisons. Cohen’s d confidence intervals were computed as d ± 1.96 × √(2/*n* + *d*^2^/(2(*n*_1_ + *n*_2_ − 2))), where n_1_ = n_2_ = 15. Extreme d values observed in HR zone comparisons are consistent with near-zero within-group variance in telemetry-derived time-series aggregates and do not indicate model misspecification.

## Results

### Reliability analysis

The reliability of time-motion analysis was established through both relative and absolute measurements, as shown in Table 1. The intra-class correlation coefficients (ICCs) for all movement categories exceeded 0.90, demonstrating excellent relative reliability. For frequency measurements, ICC values ranged from 0.91 (moderate-intensity shuffling) to 0.98 (sprinting), while for time spent in each movement category, values ranged from 0.92 (standing) to 0.97 (high-intensity shuffling). The absolute reliability, measured by coefficient of variation (CV%), was also strong, with all values below 5%, ranging from 1.1% (high-intensity shuffling frequency) to 4.6% (jumping frequency). The excellent relative reliability (ICC >  0.90) and strong absolute reliability (CV < 5%) observed across all movement categories are consistent with reliability standards established for other sport-specific performance tests (Dhahbi et al., 2016).

### Physical demands according to playing position and playing level

Physical demands differed significantly by position and level (Table 2). Guards performed 84.1 ± 28.3 high-intensity shuffling movements compared to forwards (79.8 ± 6.8) and centers (68.2 ±1.5), while centers executed 47.5 ± 1.4 jumps *versus* guards (30.9 ± 1.6) and forwards (41.9 ± 1.5). International players completed 224.0 ± 5.1 total high-intensity activities compared to national players (214.1  ± 5.4). Two-way ANOVA revealed significant main effects for position and level, with minimal interaction effects.

**Table 2:** Movement patterns and physiological responses by playing position and competitive level in U-19 female basketball players. Descriptive statistics for primary outcomes across lunar phases. Comparison of physical activity patterns and physiological responses during match-play according to playing position (guards, forwards, centers) and competitive level (international and national). Data are presented as mean ± standard deviation. Movement variables include frequencies and relative live-time spent in high-intensity activities. Physiological measures include blood lactate concentration and heart rate responses. Position, level, and interaction effects were assessed using two-way ANOVA, with effect sizes reported as eta squared (*η*^2^).

**Variable**	**Guards**	**Forwards**	**Centers**	**International**	**National**	**Position effect**	**Level effect**	**Interaction effect**
**Movement frequency (n)**								
Sprinting	59.8 ± 6.2^a^^,^^c^	62.6 ± 4.8^b^^,^^c^	56.7 ± 10.2	65.8 ± 1.6^d^	52.3 ± 5.8	*F* = 6.19 *p* = 0.007 *η*^2^ =0.34	*F* = 149.61 *p* = 0.001 *η*^2^ = 0.86	*F* = 0.45 *p* = 0.642 *η*^2^ =0.04
High-intensity shuffling	84.1 ± 28.3^a^^,^^c^	79.8 ± 6.8^b^^,^^c^	68.2 ± 1.5	91.4 ± 18.7^d^	68.4 ± 7.3	*F* = 142.35 *p* =0.001 *η*^2^ = 0.92	*F* = 79.40 *p* = 0.001 *η*^2^ = 0.77	*F* = 2.06 *p* = 0.149 *η*^2^ = 0.15
Jumping	30.9 ± 1.6	41.9 ± 1.5^b^	47.5 ± 1.4^a^^,^^b^	40.2 ± 7.7^d^	39.3 ± 8.0	*F* = 84.15 *p* =0.001 *η*^2^ = 0.88	*F* = 3.92 *p* = 0.059 *η*^2^ = 0.14	*F* = 0.61 *p* = 0.553 *η*^2^ =0.05
Static high-intensity actions	17.1 ± 3.4	25.5 ± 3.0^b^	52.1 ± 3.9^a^^,^^b^	34.5 ± 16.1^d^	28.7 ± 15.4	*F* = 236.71 *p* =0.001 *η*^2^ = 0.95	*F* = 19.25 *p* = 0.001 *η*^2^ = 0.45	*F* = 1.42 *p* = 0.261 *η*^2^ =0.11
Total high-intensity	223.3 ± 4.8^a^^,^^c^	222.0 ± 6.5^b^^,^^c^	215.5 ± 6.5	224.0 ± 5.1^d^	214.1 ± 5.4	*F* = 5.79 *p* = 0.009 *η*^2^ =0.33	*F* = 47.26 *p* = 0.001 *η*^2^ = 0.66	*F* = 0.14 *p* = 0.872 *η*^2^ =0.01
**% Live time**								
Sprinting	3.9 ± 0.3^a^^,^^c^	3.9 ± 0.4^b^^,^^c^	3.8 ± 0.5	4.2 ± 0.1^d^	3.5 ± 0.1	*F* = 0.17 *p* = 0.843 *η*^2^ =0.01	*F* = 217.78 *p* = 0.001 *η*^2^ = 0.90	*F* = 0.06 *p* = 0.946 *η*^2^ =0.01
High-intensity shuffling	4.3 ± 0.4^a^^,^^c^	4.2 ± 0.4^b^^,^^c^	4.0 ± 0.4	4.5 ± 0.1^d^	3.7 ± 0.1	*F* = 64.81 *p* =0.001 *η*^2^ = 0.84	*F* = 251.56 *p* = 0.001 *η*^2^ = 0.91	*F* = 0.16 *p* = 0.856 *η*^2^ =0.01
Jumping	2.7 ± 0.1	2.8 ± 0.1^b^	2.9 ± 0.1^a^^,^^b^	2.9 ± 0.1^d^	2.7 ± 0.1	*F* = 51.73 *p* =0.001 *η*^2^ = 0.81	*F* = 95.39 *p* = 0.001 *η*^2^ = 0.80	*F* = 0.53 *p* = 0.594 *η*^2^ =0.04
Total high-intensity	12.9 ± 1.3^a^^,^^c^	13.2 ± 1.2^b^^,^^c^	13.1 ± 1.2	14.0 ± 0.5^d^	12.1 ± 0.4	*F* = 0.89 *p* = 0.425 *η*^2^ =0.07	*F* = 89.14 *p* = 0.001 *η*^2^ = 0.79	*F* = 0.09 *p* = 0.913 *η*^2^ =0.01
**Blood lactate [La] (mmol L^−1^)**								
Mean	4.64 ± 0.12	4.93 ± 0.13^b^	5.22 ± 0.15^a^^b^	5.07 ± 0.29^d^	4.73 ± 0.29	*F* = 37.51 *p* =0.001 *η*^2^ = 0.76	*F* = 107.36 *p* = 0.001 *η*^2^ = 0.82	*F* = 1.01 *p* = 0.379 *η*^2^ = 0.08
**Heart rate**								
Average HR (bpm)	169.2 ± 3.4	169.8 ± 3.2	170.3 ± 3.3	172.5 ± 1.2^d^	166.9 ± 1.0	*F* = 4.13 *p* = 0.029 *η*^2^ =0.26	*F* = 196.92 *p* = 0.001 *η*^2^ = 0.89	*F* = 0.05 *p* = 0.954 *η*^2^ =0.00
%HRmax	86.2 ± 1.2	86.2 ± 1.2	86.3 ± 1.2	87.4 ± 0.1^d^	85.2 ± 0.1	*F* = 3.76 *p* = 0.038 *η*^2^ =0.24	*F* = 365.71 *p* = 0.001 *η*^2^ = 0.94	*F* = 0.14 *p* = 0.867 *η*^2^ =0.01

**Notes.**

a Significantly different from forwards (*p* < 0.05).

b Significantly different from guards (*p* < 0.05).

c Significantly different from centers (*p* <  < 0.05)

d Significantly different from national-level players (*p* < 0.05).

### Movement frequencies and high-intensity activities

International-level players (ILP) performed significantly more high-intensity activities than national-level players (NLP) during games (F(1,24) = 47.26, *p* < 0.001, *η*^2^ = 0.66). Position-specific analysis revealed that guards performed more high-intensity specific movements (F(2,24) = 142.35, *p* < 0.001, *η*^2^ = 0.92) than forwards and centers. In contrast, centers executed more static high-intensity actions (positioning and picks) than other positions (F(2,24) = 236.71, *p* < 0.001, *η*^2^ = 0.95) (Table 2).

Guards performed significantly more shuffling movements across all intensity levels compared to other positions (F(2,24) = 153.84, *p* < 0.001, *η*^2^ = 0.93). Centers engaged in significantly more jumping activities than guards and forwards (F(2,24) = 84.15, *p* < 0.001, *η*^2^ = 0.88). The total frequency of high-intensity activities showed significant differences across playing positions, with guards performing the highest frequency, followed by forwards and centers (F(2,24) = 5.79, *p* = 0.009, *η*^2^ = 0.33).

### Time spent in different intensity activities

The percentage of live time spent in high-intensity activities was significantly higher for ILP compared to NLP (F(1,24) = 89.14, *p* < 0.001, *η*^2^ = 0.79). Guards spent significantly more time in high-intensity shuffling movements (F(2,24) = 64.81, *p* < 0.001, *η*^2^ = 0.84) compared to forwards and centers. In contrast, centers allocated more time to jumping activities and static exertions (F(2,24) = 51.73, *p* < 0.001, *η*^2^ = 0.81) than other positions.

**Figure 1:**
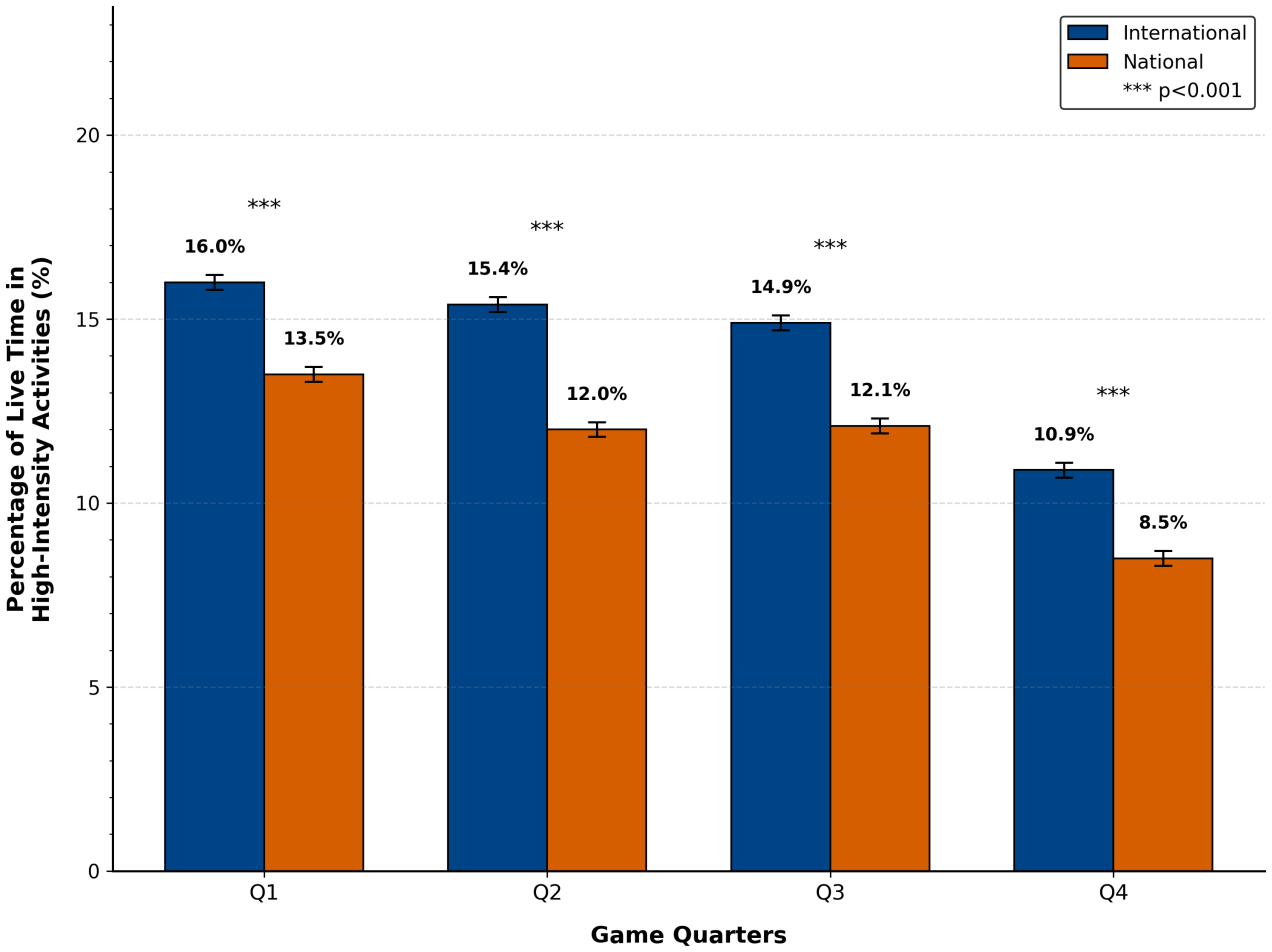
Changes in high-intensity activity across game quarters by competitive level. Percentage of live time spent in high-intensity activities across the four game quarters (Q1–Q4) in international- and national-level U-19 female basketball players. Bars represent mean values and error bars indicate standard deviation. International-level players consistently spent a greater proportion of time performing high-intensity activities compared with national-level players across all quarters. A significant decline in high-intensity activity was observed from Q1 to Q4 in both groups. *** *p* < 0.001 between competitive levels within the same quarter.

Analysis of high-intensity activities by quarter revealed significant differences across the game, with a progressive decline from Q1 to Q4 (F(3,84) = 426.89, *p* < 0.001, *η*^2^ = 0.94). This decline was more pronounced for NLP compared to ILP, particularly in Q4 (F(3,84) = 16.78, *p* < 0.001, *η*^2^ = 0.37) (Fig. 1).

### Physiological responses according to playing position and playing level

#### Heart rate responses

Heart rate data demonstrated significant differences between playing levels, with ILP maintaining higher average HR (172.5  ± 1.2 *vs.* 166.9 ± 1.0 bpm, F(1,24) = 196.92, *p* < 0.001, *η*^2^ = 0.89) and %HRmax (87.4 ±  0.1% *vs.* 85.2 ± 0.1%, F(1,24) = 365.71, *p* < 0.001, *η*^2^ = 0.94) than NLP.

**Figure 2:**
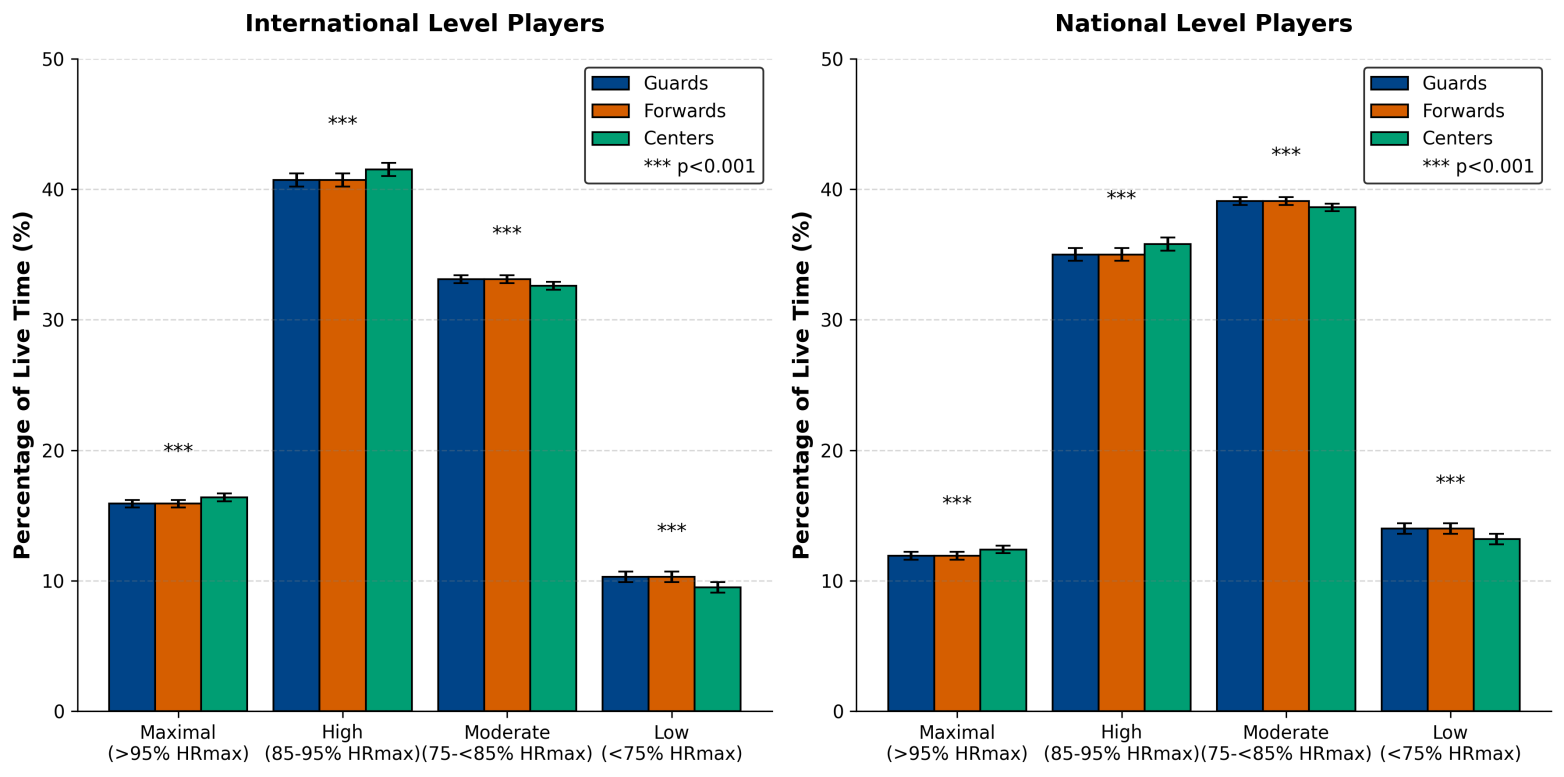
Distribution of heart rate intensity zones by playing position and competitive level. Percentage of live playing time spent in different heart rate (HR) intensity zones by guards, forwards, and centers during match-play in international-level (left panel) and national-level (right panel) U-19 female basketball players. HR zones are expressed relative to individual maximal heart rate (HRmax): maximal (>95% HRmax), high (85–95% HRmax), moderate (75–85% HRmax), and low (<75% HRmax). Bars represent mean values and error bars indicate standard deviation. Significant differences between playing positions within the same competitive level are indicated by *** (*p* < 0.001).

Time spent in different HR intensity zones varied significantly between playing levels (F(3,84) = 178.35, *p* < 0.001, *η*^2^ = 0.86), with ILP spending more time in maximal (16.1  ± 0.3% *vs.* 12.1 ± 0.3%, *p* < 0.001, *d* = 13.33 95% CI [9.76–16.90]) and high (40.9 ± 0.5% *vs.* 35.2 ± 0.5%, *p* < 0.001, *d* = 11.40 95% CI [8.33–14.47]) intensity zones than NLP. Conversely, NLP spent more time in moderate (38.9 ± 0.3% *vs.* 32.9  ± 0.3%, *p* < 0.001, *d* = 20.00 95% CI [14.71–25.29]) and low (13.6 ± 0.4% *vs.* 10.0 ± 0.4%, *p* < 0.001, *d* = 9.00 95% CI [6.54–11.46]) intensity zones. These extreme d values are arithmetically derived from very low within-group variance in telemetry-based HR zone data and should be interpreted in the context of the small subgroup size (*n* = 5 per cell) rather than as population-level effect magnitude estimates. Position-specific analysis revealed that centers maintained marginally higher percentages of HRmax compared to guards and forwards, although these differences were relatively small (F(2,24) = 4.13, *p* = 0.029, *η*^2^ = 0.26) (Fig. 2).

#### Blood lactate concentration [La]

Blood lactate concentrations varied significantly across quarters (F(3,84) = 394.62, *p* < 0.001, *η*^2^ = 0.93), with the highest values observed in Q4, followed by Q2, Q3, and Q1. ILP demonstrated significantly higher [La] values than NLP across all quarters (F(1,24) = 107.36, *p* < 0.001, *η*^2^ = 0.82).

**Figure 3:**
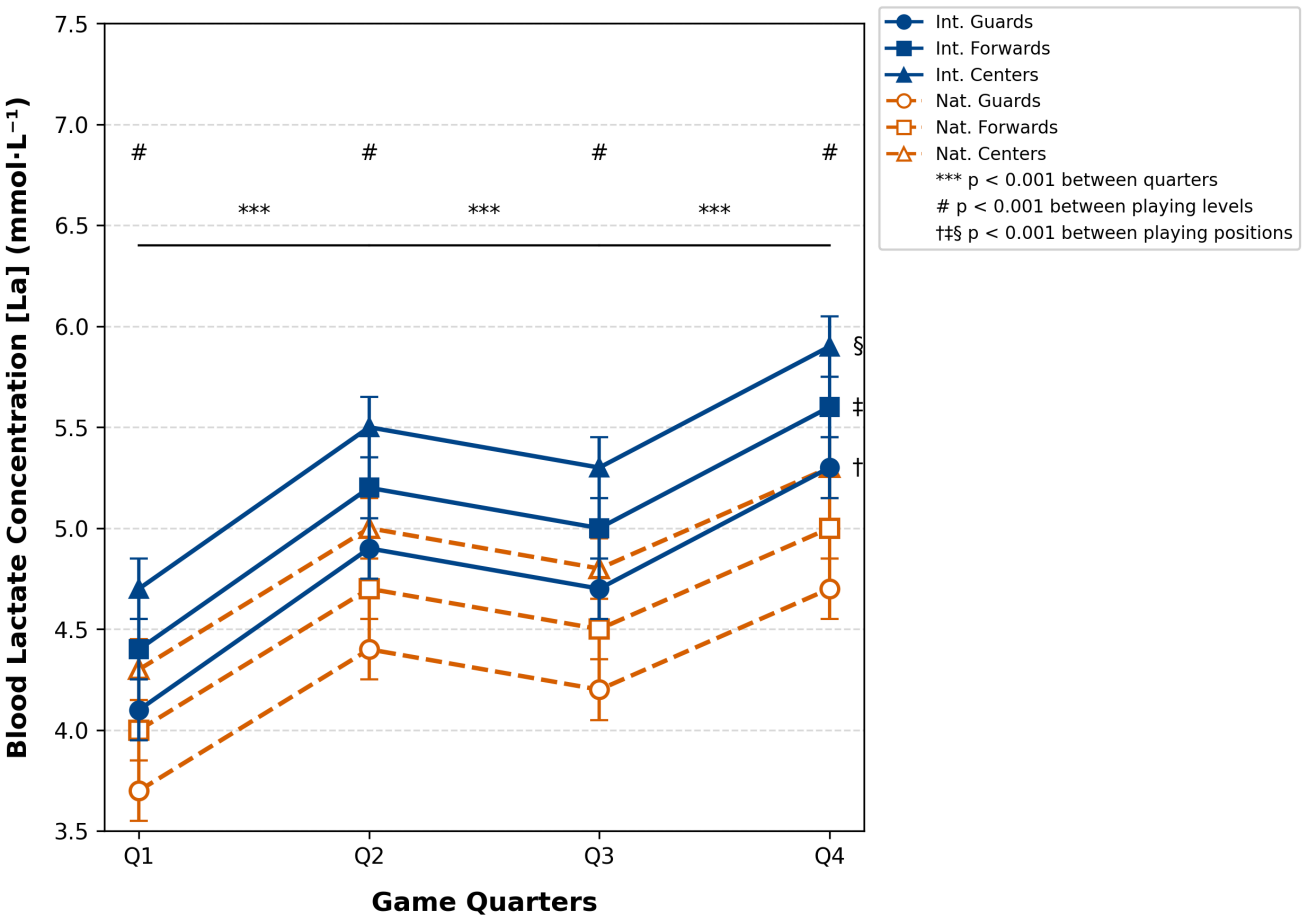
Blood lactate responses across game quarters by playing position and competitive level. Changes in blood lactate concentration ([La], mmol L^1^) across the four game quarters (Q1–Q4) in U-19 female basketball players, stratified by playing position (guards, forwards, centers) and competitive level (international *vs.* national). Data are presented as mean values with error bars indicating standard deviation. International-level players exhibited consistently higher lactate concentrations than national-level players across all quarters, with progressive increases toward the final quarter. Symbols indicate significant differences between quarters, playing positions, and competitive levels (*p* < 0.05).

Position-specific analysis revealed significant differences in [La] (F(2,24) = 37.51, *p* < 0.001, *η*^2^ = 0.76), with centers exhibiting the highest values (5.22 ± 0.15 mmol L^−1^), followed by forwards (4.93 ± 0.13 mmol L^−1^) and guards (4.64 ± 0.12 mmol L^−1^) (Table 3) (Fig. 3).

**Table 3:** Changes in high-intensity activity and blood lactate responses across game quarters by competitive level. Quarter-by-quarter comparison of high-intensity activity (% of live time) and blood lactate concentration ([La]) during match-play in international- and national-level U-19 female basketball players. Data are presented as mean ± standard deviation. Main effects of competitive level, game quarter, and their interaction were analyzed using two-way ANOVA, with effect sizes reported as eta squared (*η*^2^). Superscript letters indicate significant differences between quarters (*p* < 0.05).

**Variable**	**Quarter**	**International**	**National**	**Level effect**	**Quarter effect**	**Interaction effect**
% High-intensity activity	Q1	16.0 ± 0.2^d^	13.5 ± 0.2^d^	*F* = 953.63 *p* < 0.001 *η*^2^ = 0.98	*F* = 426.89 *p* < 0.001 *η*^2^ = 0.94	*F* = 16.78 *p* < 0.001 *η*^2^ = 0.37
	Q2	15.4 ± 0.2^c^^,^^d^	12.0 ± 0.2^c^^,^^d^			
	Q3	14.9 ± 0.2^b^^,^^d^	12.1 ± 0.2^b^^,^^d^			
	Q4	10.9 ± 0.2^a^^,^^b^^,^^c^	8.5 ± 0.2^a^^,^^b^^,^^c^			
[La] (mmol L^−1^)	Q1	4.3 ± 0.3^b^^,^^c^^,^^d^	3.9 ± 0.3^b^^,^^c^^,^^d^	*F* = 107.36 *p* < 0.001 *η*^2^ = 0.82	*F* = 394.62 *p* < 0.001 *η*^2^ = 0.93	*F* = 4.02 *p* = 0.010 *η*^2^ = 0.13
	Q2	5.1 ± 0.3^a^^,^^c^^,^^d^	4.7 ± 0.3^a^^,^^c^^,^^d^			
	Q3	4.9 ± 0.3^a^^,^^b^^,^^d^	4.5 ± 0.3^a^^,^^b^^,^^d^			
	Q4	5.5 ± 0.3^a^^,^^b^^,^^c^	5.0 ± 0.3^a^^,^^b^^,^^c^			

**Notes.**

a Significantly different from Q1 (*p* < 0.05).

b Significantly different from Q2 (*p* < 0.05).

c Significantly different from Q3 (*p* < 0.05).

d Significantly different from Q4 (*p* < 0.05).

## Discussion

This study provides the first quantitative characterization of physical and physiological demands in elite African U-19 female basketball, with concurrent assessment of movement patterns, HR responses, and metabolic markers across playing positions, competitive levels, and game quarters. Our findings establish position-specific reference profiles (guards: lateral movement specialists; centers: static exertion and jumping emphasis) and competitive-level benchmarks (international players: superior fatigue resistance) that address critical knowledge gaps in female youth basketball demand profiling. Exercise intensity and physiological responses differed substantially by position and level, with distinct patterns informing targeted conditioning approaches.

### Analysis by playing position

Position-specific analysis revealed distinct activity profiles reflecting the tactical roles and physical demands of different playing positions. Guards performed significantly more high-intensity specific movements than forwards and centers, while centers executed significantly more static high-intensity actions. These findings align with previous studies in U-19 Tunisian elite male (Ben Abdelkrim et al., 2010b; Ben Abdelkrim, El Fazaa & El Ati, 2007) and adult Spanish female basketball players (Ben Abdelkrim et al., 2010b; Ben Abdelkrim, El Fazaa & El Ati, 2007). The higher movement frequencies observed in guards likely reflect their tactical responsibility to control game pace, initiate offensive plays, and apply defensive pressure, which require frequent acceleration and deceleration (Ben Abdelkrim et al., 2009).

Position-specific sprint analysis revealed no differences across positions (F(2,24) = 0.17, *p* = 0.843, *η*^2^ = 0.01), with guards, forwards, and centers spending similar proportions of live time sprinting (3.9%, 3.9%, and 3.8%, respectively). Similar patterns have been observed among Australian female players, in which forwards spent more time sprinting than guards during games, though this difference was not significant (Scanlan et al., 2012). These patterns likely reflect tactical responsibilities, with forwards frequently covering longer distances during critical game moments such as fast breaks (Arslan et al., 2022).

However, adult Spanish female players (Espasa-Labrador et al., 2024) showed that guards spent more time sprinting than power forwards and centers (1.3 ± 0.9% *vs.* 0.2 ±0.6% and 0.3 ± 0.3%, respectively). In contrast, Tunisian U-19 males demonstrated position-specific differences, with guards performing more high-speed running than forwards and centers (Scanlan, Dascombe & Reaburn, 2011). These inconsistencies across populations likely reflect interactions between competitive level, tactical strategies, and sex-specific physiological characteristics rather than indicating position-independent sprint demands in U-19 females.

Our analysis of running patterns revealed unexpected similarities across playing positions, with no significant differences in the percentage of live-time spent running. This contradicts conventional position-specific expectations, particularly for centers, and partially aligns with previous research in men’s basketball, which reports running time percentages of 14.9%, 18.6%, and 16.4% for guards, forwards, and centers, respectively (Morrison et al., 2022). The elevated running time for centers (∼21%) observed in our study challenges the traditional understanding of center positioning. While centers typically operate as spatial anchors near the basket in both offensive and defensive schemes, contemporary basketball tactics may be evolving toward more mobile center play, particularly in youth basketball. This could reflect tactical adaptations that require centers to engage more actively in pick-and-roll actions and transition plays, moving beyond traditional low-post positioning (Reina, García-Rubio & Ibáñez, 2020).

Analysis of recovery patterns revealed position-specific differences: guards spent less time in standing and walking activities than frontcourt players. However, these differences did not reach statistical significance (*p* = 0.425). This trend suggests distinct recovery strategies across playing positions, potentially reflecting the intermittent nature of positional demands. The predominance of passive recovery (standing/walking, ∼32.5%) over active recovery methods (jogging, ∼11.5%) suggests that recovery during gameplay is primarily passive across all positions. This finding contrasts with observations in male U-19 basketball (Ben Abdelkrim, El Fazaa & El Ati, 2007) and adult female basketball (Morrison et al., 2022), where centers typically spend more time in recovery activities than other positions. These differences may reflect gender-specific tactical approaches or developmental aspects unique to female U-19 basketball in North Africa.

Our findings reveal that U-19 female basketball players spent approximately 26–28% of live game time in specific movement patterns (high, moderate, and low-intensity shuffling). This is notably lower than the ∼41% reported in U-19 male basketball (Ben Abdelkrim, El Fazaa & El Ati, 2007), highlighting potential gender differences in movement emphasis. Position-specific analysis of shuffling movements showed that guards performed significantly more high-intensity shuffling than other positions (*p* < 0.001, *η*^2^ = 0.84), with large effect sizes underscoring the practical significance of these differences. This position-specific pattern aligns with previous research (Scanlan, Dascombe & Reaburn, 2011) and reflects the tactical responsibilities of guards, who frequently engage in defensive actions that require lateral movement, particularly when defending ball handlers. The absence of positional differences in low-intensity shuffling suggests that specific fundamental defensive movements are consistent across positions, while high-intensity defensive actions are more position-specialized (Ben Abdelkrim, El Fazaa & El Ati, 2007).

Position-specific analysis of basketball-specific movements revealed distinct patterns in jumping and static actions that reflect specialized positional roles. Centers performed significantly more jumps than other positions (*p* < 0.001, *η*^2^ = 0.88), consistent with their primary responsibility for rebounding and rim protection. This increased jumping frequency likely increases neuromuscular demands in centers compared to other positions. Our findings align with research on Spanish female adult basketball (Koo & Li, 2016), which reported that centers spend more time jumping (3.1%) compared to guards (1.1%) and forwards (1.9%). Similarly, centers in our study engaged in significantly more static high-intensity actions (positioning and picks) than forwards and guards (*p* < 0.001, *η*^2^ = 0.95), a pattern also observed in Spanish adult female basketball (Reina, García-Rubio & Ibáñez, 2020). These consistent position-specific patterns across different populations suggest fundamental position-based role specialization that transcends age groups and geographic regions in women’s basketball.

Analysis of physiological responses revealed that heart rate was minimally influenced by playing position, with all positions maintaining similar cardiovascular loads during gameplay (*p* = 0.038, *η*^2^ = 0.24). This finding contrasts with previous research on Spanish adult females (Koo & Li, 2016) and Australian female basketball players (Hopkins, Schabort & Hawley, 2001), where guards typically exhibited higher heart rates than players in other positions. Similarly, U-19 Tunisian male players showed position-specific differences in heart rate responses (Ben Abdelkrim et al., 2010b). The absence of substantial position-based heart rate differences in our study could indicate that U-19 female basketball in North Africa involves more balanced cardiovascular demands across positions, potentially reflecting differences in tactical approaches or developmental strategies compared to adult basketball or male youth basketball.

The high HR response (*i.e.,* in the high HR zone) for forwards and centres was expected, given their considerable involvement in total high-intensity activities. Several authors (Ben Abdelkrim, El Fazaa & El Ati, 2007; Rodríguez-Alonso et al., 2003) have proposed that such activities account for a significant portion of basketball’s energy demand. Moreover, it appears that the considerable bouts of static activity in which centres are engaged, such as picking, blocking, and maintaining position against the physical resistance of an adversary, have led to their critical HR responses. Almost all muscles are recruited during such movements, both in isometric and dynamic activities, which significantly affects heart rate (Sallet et al., 2005). However, this interpretation should be carefully considered, as HR is influenced by other variables, such as thermal factors, hydration status, psychological arousal, emotional stress, and mental pressure (Stojanović et al., 2018).

Blood lactate concentration revealed significant position-specific metabolic demands, with centers exhibiting the highest values (5.22 mmol L^−1^), followed by forwards (4.93 mmol L^−1^) and guards (4.64 mmol L^−1^) (*p* < 0.001, *η*^2^ = 0.76). This metabolic pattern contrasts with movement-based findings, suggesting that static high-intensity actions, predominant in centers, impose substantial anaerobic metabolic demands despite appearing less dynamic. The higher blood lactate concentrations in centers likely reflect their engagement in repeated jumping and static exertions such as screening, positioning, and rebounding contests (Ziv & Lidor, 2009), which involve significant isometric muscle actions known to accelerate lactate production. These position-specific metabolic differences highlight the importance of considering both dynamic movements and static exertions when evaluating basketball demands and developing position-specific training interventions. As noted in previous research (Ziv & Lidor, 2009), blood lactate measurements reflect recovery activities, individual aerobic capacity influences activities performed minutes before sampling, and removal rates (Hargreaves & Spriet, 2020). These factors should be considered when interpreting these findings.

### Analysis by player’s level

Our analysis of competitive-level differences revealed that international-level players (ILP) demonstrated significantly higher game demands than national-level players (NLP), extending similar observations previously reported in male U-19 basketball (Ben Abdelkrim et al., 2010a). The substantial difference in high-intensity activity across competitive levels (*p* < 0.001, *η*^2^ = 0.66) is a key finding with important practical implications. These differences likely stem from the increased movement complexity in higher-level competition, which requires more frequent transitions between movement categories (such as starting, stopping, and changing direction) and, consequently, greater energy expenditure to overcome movement inertia repeatedly (Gottlieb, Shalom & Calleja-Gonzalez, 2021). The greater frequency of high-intensity efforts observed in ILP within this playoff context is consistent with between-level differences previously reported in male U-19 basketball (Ben Abdelkrim et al., 2010a), though a single-game design precludes attribution to stable physiological adaptation.

Competitive level differences were evident across multiple physiological and performance metrics. International players demonstrated significantly greater engagement in high-intensity activities (*p* < 0.001, *η*^2^ = 0.79) coupled with elevated physiological responses, including higher blood lactate concentrations (*p* < 0.001, *η*^2^ = 0.82) and heart rate values (*p* < 0.001, *η*^2^ = 0.89). Notably, the distribution of time spent in heart rate intensity zones differed: international players spent substantially more time in the maximal and high-intensity zones than their national counterparts. These findings align with previous research in basketball and other team sports (Rodríguez-Alonso et al., 2003; Stojanović et al., 2018), confirming that higher competitive levels impose greater physiological demands. The superior ability of international players to perform at higher intensities likely reflects a combination of factors, including enhanced physical conditioning, greater tactical proficiency, and potentially genetic factors that may influence physiological capacity and trainability.

Our analysis of recovery patterns revealed intriguing competitive-level differences that may contribute to performance sustainability. International players appear to use recovery periods more strategically between high-intensity efforts, optimizing the balance between work and recovery. This more pronounced intermittent activity pattern likely contributes to the higher blood lactate concentrations observed in international players, as passive recovery periods (standing) are less effective for lactate clearance than active recovery (walking or jogging) (Ardigò et al., 2020). These findings highlight the importance of not only conditioning players to perform high-intensity actions but also training them to optimize recovery strategies during gameplay, particularly as players advance to higher competitive levels.

Our quarter-by-quarter analysis revealed significant fatigue-related performance decrements across game duration for both playing levels (*p* < 0.001, *η*^2^ = 0.94), with ILP maintaining higher high-intensity activity levels across all quarters than NLP. The smaller Q1-to-Q4 decline in ILP (16.0% to 10.9%; 31.9%) compared to NLP (13.5% to 8.5%; 37.0%) (*p* < 0.001, *η*^2^ = 0.37) indicates a within-game performance difference in this playoff context, consistent with greater within-game performance sustainability in ILP. Whether this reflects stable physiological superiority or context-dependent factors cannot be determined from a single-game design.

These competitive-level differences in fatigue resistance align with findings from other team sports (Morrison et al., 2022) and highlight the critical importance of developing not only the capacity to perform high-intensity actions but also the ability to sustain this performance throughout an entire game. The higher estimated V̇O_2_max in ILP (53.73 ml kg^−1^ min^−1^) *versus* NLP (50.59 ml kg^−1^ min^−1^) is consistent with the greater within-game high-intensity activity observed, though the direction of this relationship cannot be established from cross-sectional data. The overall physiological demands observed in our study align with those reported in previous research on elite female basketball (Sallet et al., 2005) and elite U-19 male basketball (Ben Abdelkrim, El Fazaa & El Ati, 2007), supporting the ecological validity of our findings.

The observed performance differentials between competitive levels may inform player evaluation frameworks. International-level players sustained higher high-intensity activity frequencies (224.0 ± 5.1 *vs.* 214.1 ± 5.4 repetitions) and spent more time in maximal HR zones (16.1 ± 0.3% *vs.* 12.1 ± 0.3%), suggesting these metrics warrant consideration in readiness assessment. However, the single-game design and small subgroup size prevent establishment of definitive selection thresholds. Future longitudinal research with larger samples is needed to validate whether these observed differences represent stable performance markers suitable for talent identification purposes.

This study presents fundamental methodological constraints that limit generalizability. First, the single-game observation design prevents assessment of within-player variability and match-to-match fluctuations in physical demands, which are influenced by contextual factors including opponent quality, game outcome, and tactical approach (Ferioli et al., 2020; Fox et al., 2018). Consequently, the observed position-specific and competitive-level differences represent snapshots within a specific competitive context (playoff games) rather than stable individual or group characteristics. Furthermore, key contextual variables including score margin, game outcome, opponent competitive strength, and possession structure were not controlled. Each of these factors is known to influence physical demands in basketball (Ferioli et al., 2020; Fox et al., 2018), and their variation across the eight analyzed games may have contributed to the observed between-level differences independently of player-level physiological characteristics. The small subgroup size (*n* = 5) further constrains statistical robustness and increases vulnerability to outlier influence and regression-to-the-mean effects. Second, the menstrual cycle phase was not controlled or documented, despite evidence that hormonal fluctuations may influence physiological responses, substrate metabolism, and perceived exertion in female athletes (Meignié et al., 2021). Third, our analysis was conducted during playoff games, which may place different demands on players than regular-season matches. Fourth, individual nutritional status was not systematically monitored. Although hydration was provided ad libitum and standardized meals were offered, variations in pre-game nutrition may have influenced physiological responses. Future research should address these limitations by incorporating longitudinal multi-game monitoring, menstrual cycle tracking, additional physical metrics (acceleration/deceleration profiles), and assessment across different competitive phases.

## Conclusions

This study establishes the first comprehensive demand profile for elite African U-19 female basketball, revealing position-specific activity patterns (guards: 84.1 ± 28.3 high-intensity shuffling actions; centers: 41.2 ± 8.7 jumps, elevated [La] 5.22 ± 0.15 mmol L^−1^) and competitive-level differences (international players: 31.9% *vs.* national: 37.0% Q1-to-Q4 performance decline). These quantitative benchmarks inform conditioning priorities: guards require lateral-movement capacity development; centers necessitate anaerobic tolerance and vertical power training; national-level players benefit from fatigue-resistance protocols targeting fourth-quarter performance maintenance. However, the single-game design and small subgroups (*n* = 5) constrain generalizability. Future research should validate these profiles through longitudinal multi-game monitoring with larger samples to establish stable position-specific and level-specific performance standards suitable for evidence-based conditioning prescription and talent development frameworks in female youth basketball.

##  Supplemental Information

10.7717/peerj.21218/supp-1 Supplemental Information 1 Raw and processed data for psychological outcomes following a four-week CrossFit intervention in first-year law students Anonymized participant-level data collected at baseline and post-intervention for the intervention and control groups, including scores for emotional balance (EBS), self-esteem (SEI), and perceived stress (PSS), as well as demographic variables used in the statistical analyses.

10.7717/peerj.21218/supp-2 Supplemental Information 2 STROBE checklist for reporting a cross-sectional study on physical and physiological demands in elite U-19 female basketball players STROBE (Strengthening the Reporting of Observational Studies in Epidemiology) checklist for cross-sectional studies, indicating where each reporting item is addressed within the manuscript. The checklist is provided to ensure transparency, methodological rigor, and compliance with reporting guidelines during peer review.

## Additional Information and Declarations

### Data Availability

The following information was supplied regarding data availability: The raw data is available in the Supplementary File.
